# Profile of dorsal root ganglion neurons: study of oxytocin expression

**DOI:** 10.1186/s13041-022-00927-6

**Published:** 2022-05-09

**Authors:** Taisei Noguri, Dai Hatakeyama, Takashi Kitahashi, Kotaro Oka, Etsuro Ito

**Affiliations:** 1 Department of Biology, Waseda University, Tokyo, 162-8480 Japan; 2 Laboratory of Biochemistry, Faculty of Pharmaceutical Sciences, Tokushima Bunri University, Tokushima, 770-8514 Japan; 3 Kushiro Nature Conservation Office, Ministry of the Environment, Kushiro, 085-8639 Japan; 4 Department of Biosciences and Informatics, Faculty of Science and Technology, Keio University, Yokohama, 223-8522 Japan; 5 Waseda Research Institute for Science and Engineering, Waseda University, Tokyo, 169-8555 Japan; 6 Graduate Institute of Medicine, School of Medicine, Kaohsiung Medical University, Kaohsiung, 80708 Taiwan

## Abstract

Although dorsal root ganglion (DRG) neurons have been so far classified according to the difference in their fibers (Aβ, Aδ, and C), this classification should be further subdivided according to gene expression patterns. We focused on oxytocin (OXT) and its related receptors, because OXT plays a local role in DRG neurons. We measured the mRNA levels of OXT, OXT receptor (OXTR), vasopressin V1a receptor (V1aR), transient receptor potential cation channel subfamily V member 1 (TRPV1), and piezo-type mechanosensitive ion channel component 2 (Piezo2) in single DRG neurons by using real-time PCR, and then performed a cluster analysis. According to the gene expression patterns, DRG neurons were classified into 4 clusters: Cluster 1 was characterized mainly by Piezo2, Cluster 2 by TRPV1, Cluster 4 by OXTR, and neurons in Cluster 3 did not express any of the target genes. The cell body diameter of OXT-expressing neurons was significantly larger in Cluster 1 than in Cluster 2. These results suggest that OXT-expressing DRG neurons with small cell bodies (Cluster 2) and large cell bodies (Cluster 1) probably correspond to C-fiber neurons and Aβ-fiber neurons, respectively. Furthermore, the OXT-expressing neurons contained not only TRPV1 but also Piezo2, suggesting that OXT may be released by mechanical stimulation regardless of nociception. Thus, mechanoreception and nociception themselves may induce the autocrine/paracrine function of OXT in the DRG, contributing to alleviation of pain.

## Introduction

The dorsal root ganglia (DRGs) convey peripheral sensory information to the central nervous system, and they are composed of several types of neurons and glial cells [1]. DRG neurons have been classically categorized by cell body size and myelinated/unmyelinated fibers. Previous reports showed that DRG neurons with large cell bodies and myelinated Aβ fibers transmit mechanoreception, whereas those with medium-sized cell bodies and myelinated Aδ fibers and those with small cell bodies and unmyelinated C fibers transmit nociception [1]. However, because a variety of gene expression patterns are observed in DRG neurons, it is now considered that the classification of DRG neurons should be further subdivided [2, 3].

On the other hand, a neuropeptide, oxytocin (OXT), was reported to play some roles in the DRGs [4–6]. OXT is released from the posterior pituitary gland and has long been known as a neuropeptide that stimulates uterine contractions to hasten childbirth and is involved in lactation [7]. However, because OXT also acts on DRG neurons and suppresses the firing of action potentials, its analgesic effects have also attracted attention [8, 9]. Expression of OXT was also confirmed in DRG neurons [10]. These facts suggest that OXT expressed in DRG neurons may locally exert a rapid analgesic effect, apart from the classic effects of OTX released from the posterior pituitary gland.

Based on the hypothesis that the expression and action of OXT differ depending on the type of DRG neuron, we examined the expression of OXT and its related receptors in DRG neurons and attempted to classify DRG neurons according to their gene expression profiles.

## Methods

### Preparation of single DRG neurons

We used male C57BL/6JJmsSlc mice (8–10 weeks old). These mice were obtained from Japan SLC and maintained in specific pathogen-free conditions in our animal facility. The isolation method of DRG neurons was modified from previous studies [11]. Briefly, 6 mice were anesthetized with sevoflurane and then decapitated. The DRGs were taken out from L1–L6 and incubated in Hanks’ balanced salt solution without Ca^2+^ and Mg^2+^ (HBSS(−)) containing 0.65 mg/mL collagenase and 3.0 mg/mL dispase for 30 min at 37 ℃. The cells were further kept in the above collagenase solution for more than 1 h at room temperature, and then dispersed in minimum essential medium (MEM) with 5% fetal bovine serum (FBS) by pipetting. The cells were incubated in MEM with 5% FBS on collagen-coated dishes for 4 h at 37 ℃ under 5% CO_2_. Fifteen DRG neurons were obtained from each mouse, but some neurons were lost. The diameter of each neuron in the dishes was measured with a micrometer equipped with a microscope (Zeiss Axio Vert.A1).

### Single-cell real-time PCR

The protocol of single-cell real-time PCR was modified from previous studies [12]. Single DRG neurons were picked up from the dishes with a micropipette and put into micro tubes. RNA extraction and reverse-transcription were performed using RT-RamDA cDNA synthesis kit (RMD-201T, Toyobo) according to the manufacturer’s instructions. We measured the expression levels (Ct values) of mRNA for OXT [13], OXT receptor (OXTR) [14], vasopressin V1a receptor (V1aR, cross-reaction with OXT) [8, 11], transient receptor potential cation channel subfamily V member 1 (TRPV1, a marker for nociceptive neurons) [15, 16], and piezo-type mechanosensitive ion channel component 2 (Piezo2, a marker for mechanoreceptive neurons) [17] by using single-cell real-time PCR. All PCR amplifications were performed using BlasTaq 2× qPCR MasterMix (G891, Applied Biological Materials) according to the manufacturer’s instructions. Briefly, PCR was performed in a total volume of 10 µL containing 1 µL of cDNA sample, 5 µL of MasterMix, 0.05 µL of a forward primer (50 µM, Table 1), 0.05 µL of a reverse primer (50 µM, Table 1) and 3.9 µL of sterilized water using StepOnePlus real-time PCR system (Applied Biosystems). GAPDH and β-actin were used as the reference genes. Relative expression levels of the target genes were calculated using the ΔCt method (ΔCt value = Ct value of target gene − averaged Ct value of the 2 reference genes). To perform cluster analysis and statistical analysis, when the results of real-time PCR of a sample showed ‘undetermined’ (i.e., the expression level was below the detection limit), we assigned 41 as the Ct value of the sample, as PCR was performed until the 40th cycle.

**Table 1:** Primers for single-cell real-time PCR

		Primer sequence (5′–3′)	Accession number
OXT	Forward	TTGGCTTACTGGCTCTGACCTC	NM_011025
	Reverse	GGGAGACACTTGCGCATATCCAG	
OXTR	Forward	TTCTTCGTGCAGATGTGGAG	NM_001081147
	Reverse	CCTTCAGGTACCGAGCAGAG	
V1aR	Forward	TGTGGTCAGTCTGGGATACC	NM_016847
	Reverse	GGGAAGCTCTGGACACAATC	
TRPV1	Forward	ATCATCAACGAGGACCCAGG	NM_001001445
	Reverse	TGCTATGCCTATCTCGAGTGC	
Piezo2	Forward	TCAGAACCAACCAAAGCAACG	NM_001039485
	Reverse	TTGTAAGCAGGTGTGATGCGG	
GAPDH	Forward	TATGACTCCACTCACGGCAAAT	NM_001289726
	Reverse	GGGTCTCGCTCCTGGAAGAT	
β-actin	Forward	GACTCATCGTACTCCTGCTTG	NM_007393
	Reverse	GATTACTGCTCTGGCTCCTAG	

### Cluster analysis

Using the Morisita-Horn index [18, 19] of the dissimilarity in the target gene expression pattern, cluster analysis of DRG neurons was performed. The Morisita-Horn index (or the Morisita’s overlap index) is a statistical measure of dispersion of individuals in a population. It is used mainly in ecology to compare overlap among samples [18, 19]. The formula is based on the assumption that increasing the size of the samples will increase the diversity because it will include different habitats. On the other hand, the index is designed to avoid the influence of the data number per cluster. Thus, it lends itself to our present cluster analysis. The distance among the clusters was determined by the Ward method [20]. Note that in the following sections, the results of the present study are discussed in terms of both gene expression patterns and neuron diameters, whereas the cluster analysis was performed based on gene expression patterns only.

### Statistics

One-way ANOVA followed by a post-hoc Scheffé test was used for comparison among multiple groups. Mann-Whitney *U* test was used for comparison between two groups. *P* < 0.05 was considered to be statistically significant. The statistics software used was R (version 4.1.2) and FreeJSTAT (version 22.0E).

## Results

### Size of isolated DRG neurons and their expression of oxytocin and its related receptors

**Fig. 1:**
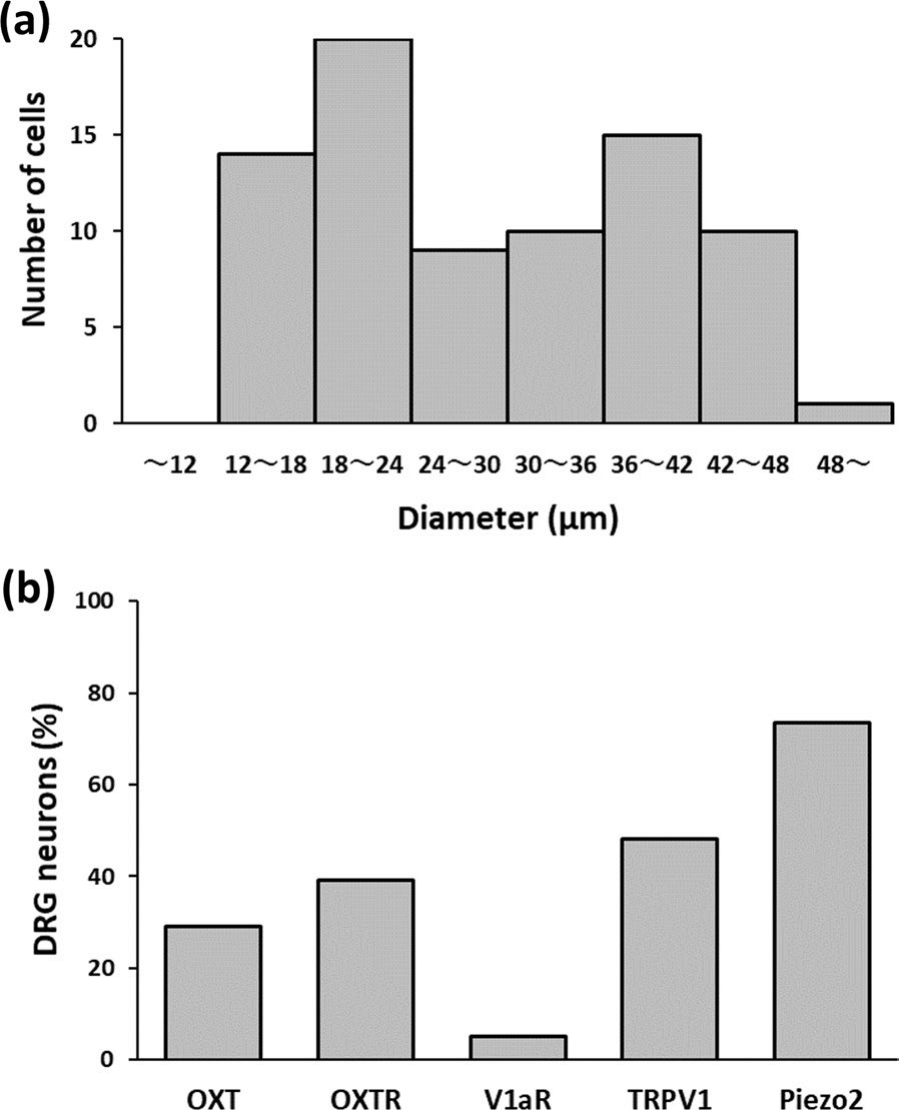
Size of isolated DRG neurons and gene expression in the cells. **a** Diameters of DRG neurons varied from 14.5 to 49.0 μm. The total number of the isolated cells was 79. **b** Percentage of DRG neurons expressing each target molecule. *OXT* oxytocin, *OXTR* OXT receptor, *V1aR* vasopressin V1a receptor, *TRPV1* transient receptor potential cation channel subfamily V member 1, *Piezo2* piezo-type mechanosensitive ion channel component 2

The diameters of single DRG neurons picked up from the culture dishes were measured with a micrometer (Fig. 1a). The diameters varied from 14.5 to 49.0 μm (n = 79). The amount of the mRNA for 5 molecules in relation to OXT, i.e., OXT, OXTR, V1aR, TRPV1, and Piezo2, was examined in single DRG neurons by using real-time PCR. When fluorescence signal strength above threshold was obtained by the 40th cycle (Ct ≦ 40), we judged that the single DRG neuron expresses the target molecule. Of 79 single DRG neurons, 58 neurons expressed Piezo2 (73%), 38 neurons TRPV1 (48%), 31 neurons OXTR (39%), 23 neurons OXT (29%), and 4 neurons V1aR (5%) (Fig. 1b).

### Cluster analysis of isolated single DRG neurons

**Fig. 2:**
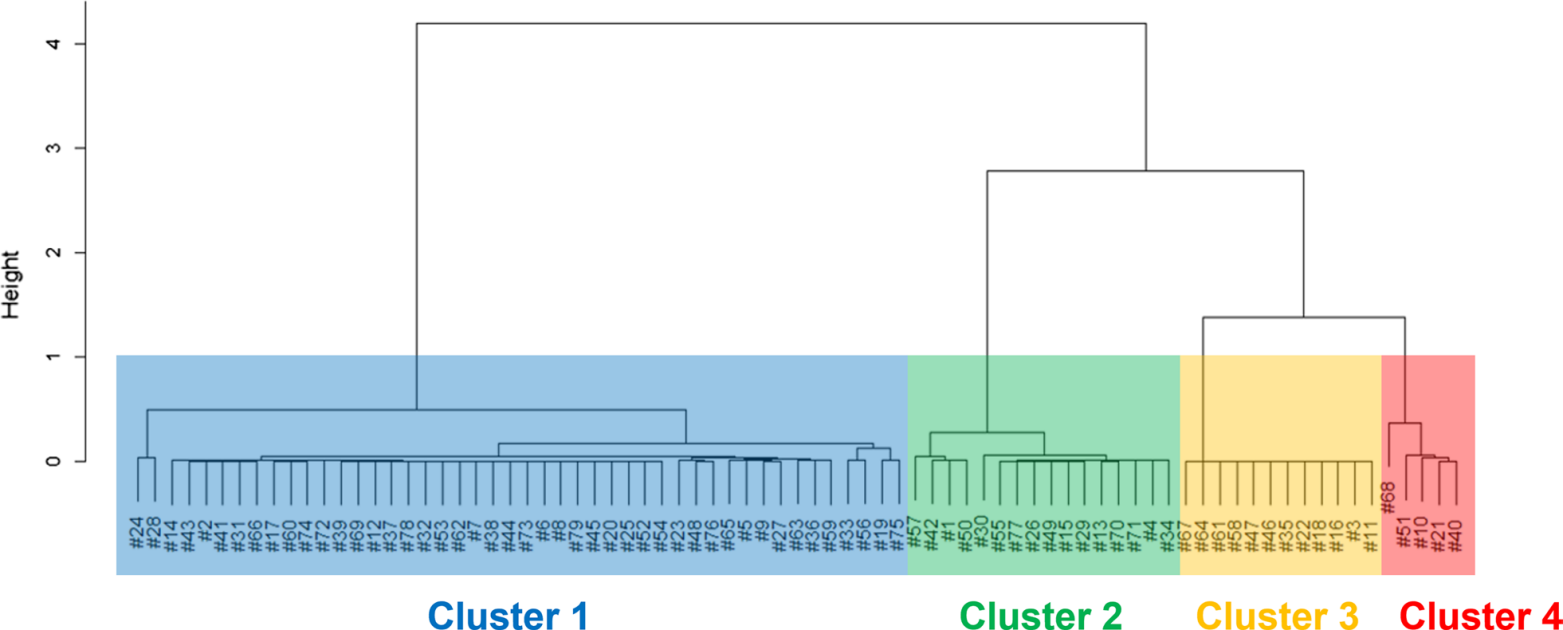
Cluster analysis for isolated DRG neurons. The neurons were classified into 4 clusters by cluster analysis of gene expression patterns using the Morisita-Horn index. Cluster 1, Cluster 2, Cluster 3, and Cluster 4 contained 46, 16, 12, and 5 cells, respectively. The height was obtained by the Ward method

A cluster analysis was performed by using the Morisita-Horn index and the Ward method (Fig. 2). Based on the gene expression pattern, DRG neurons were classified into 4 groups. Of 79 single DRG neurons, Cluster 1 contained 46 neurons (59%), Cluster 2 contained 16 neurons (20%), Cluster 3 contained 12 neurons (15%), and Cluster 4 contained 5 neurons (6%).

### Cell body size and expression of oxytocin and its related receptors in the 4 clusters

**Fig. 3:**
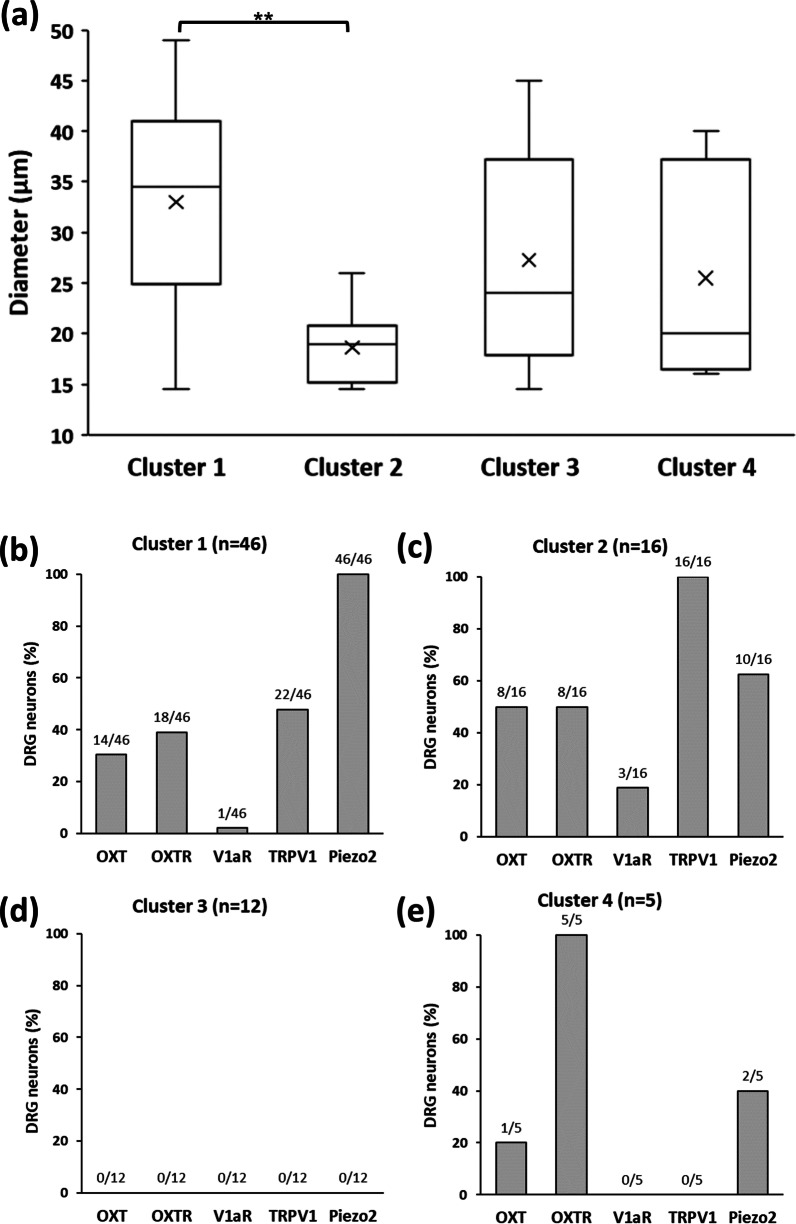
Characterization of the 4 clusters. **a** Diameters of DRG neurons in each cluster are expressed in a box plot. **P < 0.01. **b**–**e** Percentage of isolated DRG neurons expressing target molecules in Cluster 1, Cluster 2, Cluster 3, and Cluster 4 are shown. The abbreviations are the same as those in Fig. 1

The characteristics of the 4 clusters were examined (Fig. 3). The median diameters of DRG neurons in Clusters 1, 2, 3, and 4 were 34.5, 19.0, 24.0, and 20.0 μm, respectively (Fig. 3a). The cell sizes of Cluster 1 DRG neurons were significantly larger than those of Cluster 2 neurons (*P* < 0.01). When focusing on the percentage of cells expressing a particular target molecule in the cluster, we found that Cluster 1 was characterized mainly by the high expression of Piezo2, Cluster 2 by TRPV1, and Cluster 4 by OXTR, whereas cells in Cluster 3 did not express any of the target genes (Fig. 3b–e).

### Comparison of characteristics between ﻿Cluster 1 and Cluster 2

**Fig. 4:**
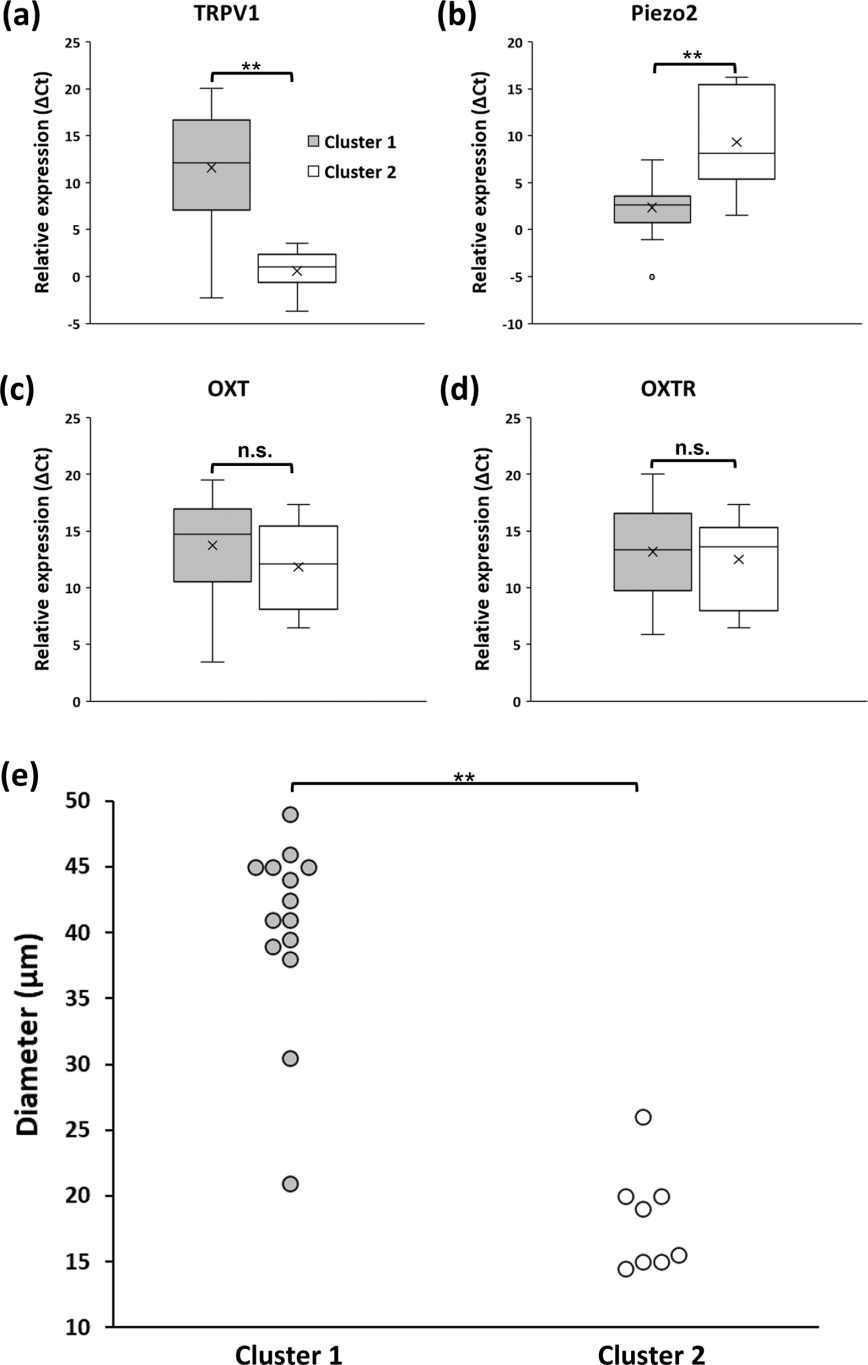
Comparison between Cluster 1 and Cluster 2. **a** Comparison of ΔCt value for TRPV1. The smaller the ΔCt value is, the larger the expression level is. **P < 0.01. **b** Comparison of ΔCt value for V1aR. **c** Comparison of ΔCt value for OXT. There is no significant difference between the 2 clusters. **d** Comparison of ΔCt value for OXTR. There is no significant difference between the 2 clusters. **e** Diameters of OXT-expressing neurons in Cluster 1 and Cluster 2. **P < 0.01

When comparing Cluster 1 and Cluster 2, the relative expression level of TRPV1 was significantly higher in Cluster 2 (Fig. 4a, *P* < 0.01). It is important to note that the smaller ΔCt is, the higher the expression level is. For Piezo2, the relative expression level in Cluster 1 was significantly higher compared to Cluster 2 (Fig. 4b, *P* < 0.01). For OXT and OXTR, there were no significant statistical differences between Cluster 1 and Cluster 2 (Fig. 4c, d). When we focused on OXT-expressing cells among DRG neurons, the average diameter of OXT-expressing neurons in Cluster 1 was 40.5 μm, indicating that OXT-expression neurons in Cluster 1 possess the large cell body (Fig. 1). On the other hand, the average diameter of OXT-expressing neurons in Cluster 2 was 18.1 μm, which was significantly smaller than that in Cluster 1 (Fig. 4e, *P* < 0.01).

### Co-expression of OXT and TRPV1 and that of OXT and Piezo2 in single DRG neurons

**Fig. 5:**
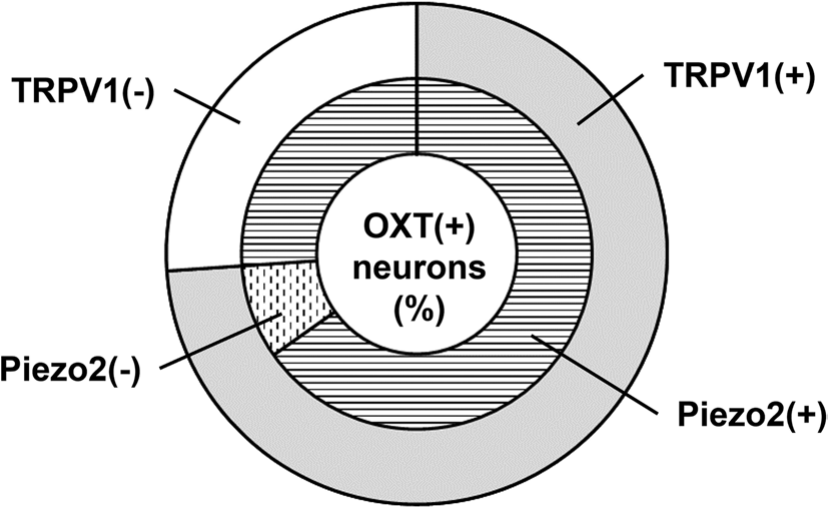
Co-expression of OXT and TRPV1 and that of OXT and Piezo2 in DRG neurons. When assuming OXT-expressing neurons was 100%, RPV1(+) and Pizo2(+) in OXT-expressing neurons was 65%; TRPV1(+) and Pizo2(−) was 9%; and TRPV1(−) and Pizo2(+) was 26%

Previous reports showed that OXT and TRPV1 were co-expressed in DRG neurons [10]. Our single-cell real-time PCR approach showed that about three fourths of DRG neurons expressing OXT also co-expressed TRPV1 (Fig. 5), reconfirming the co-expression of OXT and TRPV1 in DRG neurons [10]. Furthermore, most OXT-expressing DRG neurons, including those that did not express TRPV1, were found to co-express Piezo2. As far as we know, the co-expression of OXT and Piezo2 was confirmed for the first time in the present study.

## Discussion

In the present study, we classified DRG neurons into 4 clusters according to the expression patterns of OXT and its related receptors using single-cell real-time PCR. Cluster 1 was characterized mainly by the high expression of Piezo2, Cluster 2 by TRPV1, and Cluster 4 by OXTR. Whereas Cluster 1 contained cells with a wide range of diameters, OXT-expressing cells were found to be the large cells in the cluster. In particular, the OXT-expressing DRG neurons with large-diameter cell bodies (Cluster 1) may correspond to neurons of Aβ fibers (mechanoreception) and the OXT-expressing DRG neurons with small-diameter cell bodies (Cluster 2) may correspond to neurons of C fibers (nociception).

The DRG neurons classified as Cluster 2 in the present study highly expressed TRPV1 and were supposed to possess unmyelinated C fibers. As it has been shown that OXT acts directly on TRPV1 [16], it is possible that the analgesic effect of OXT is exerted by alleviating the perception of pain transmitted through the activation of DRG neurons in Cluster 2.

In the present study, some cells in Cluster 2 showed co-expression of V1aR and TRPV1. Han and colleagues showed that OXT significantly increased potassium conductance via V1a receptors in DRG neurons using the whole cell patch clamp recording. They considered that analgesic effects produced by peripheral administration of OXT were attributable to the activation of V1a receptors, resulting in reduction of TRPV1 activity and enhancement of potassium conductance in DRG neurons [11]. Thus, the analgesic effects of OTX via activation of V1aR may occur in DRG neurons that are classified as Cluster 2.

In terms of peripheral OXT secretion, our results demonstrated that OXT-expressing neurons contain Piezo2 in addition to TRPV1 for the first time, suggesting that not only nociception but also mechanical stimulation can induce OXT secretion in the DRGs. Taken together, in the DRGs, OXT should be released by mechanoreception and nociception, acting on non-myelinated C fibers to relieve pain. The pain-relief effects of massage or patch adhesion [21] could be via this peripheral action of OXT.

The following results may provide some suggestions about the peripheral action of OXT. Saito and colleagues showed the effectiveness for 300 patients of pyramidal thorn patch adhesion on pain regions as a complementary medicine, resulting that patch adhesion can induce pain relief [21]. Their interpretation for the effects of patch adhesion was as follows. The pathological pain signal appears in the normal peripheral tissue and in nerves that activate Aδ fiber high-threshold mechanoreceptors and C fibers [22]. This pain signal is considered to be reduced by gentle mechanical stimulation of the skin (e.g., application of pyramidal thorn patches) that activates Aβ fiber low-threshold mechanoreceptors [22, 23]. The interaction between the nociceptive signal (Aδ and C fibers) and the non-nociceptive signal (Aβ fibers) was introduced as the gate control theory of pain [24, 25]. The gate control theory hypothesizes that non-nociceptive input closes the gates to nociceptive input, which prevents pain sensations from traveling to the central nervous system. Therefore, Aβ fibers are considered to inhibit the effects of the firing of Aδ and C fibers. Our present findings suggested that Cluster 1 DRG neurons, probably corresponding to Aβ-fiber neurons, may release OXT following to mechanical stimulation regardless of nociception. That is, mechanical stimulation is considered to release OXT, resulting in alleviation of pain.

As described in Introduction, OXT is well known to be also synthesized and to function in the brain. OXT is synthesized in neurons of the supraoptic nucleus and paraventricular nucleus of the hypothalamus after specific stimulation of the brain. These neurons project to the posterior pituitary, where OXT is released into the blood for delivery to peripheral tissues as well as into the brain. Many researchers have strongly believed that the effect of OXT on pain relief can be controlled by this central OXT. In the brain, OXTergic antinociception is thought to be mediated by GABAergic interneurons that inhibit the primary nociceptive inputs conveyed by Aδ and C fibers to the spinal cord [26, 27]. The involvement of GABA mediated by OXT in pain relief was also confirmed in newborn rats [28]. Furthermore, the involvement of V1aR was found using knock-out mice, because OXTR knock-out mice displayed a pain phenotype identical to wild-type mice, whereas OXT-induced analgesia was completely absent in V1aR knock-out mice [29]. These mysterious phenomena may be caused by an unknown cross-talk reaction between the OXT and V1a systems.

Previous studies have suggested that humans and other mammals feel comfort (i.e., pleasure) when the perception of mild skin stimulation is sent to the brain via C fibers [30, 31]. It is possible that, in such a situation, OXT is released not only from the hypothalamus but also in the DRG. It will be interesting to see how the central and local actions of OXT interact with each other in future studies.

About the cell size of DRG neurons, the researchers discuss it through relative comparisons, using the terms like large diameter or small diameter. For example, measuring the cell size in stained tissues [32] and measuring the cell size after isolating in our present study are fairly different. The size also depends on the development of mouse. In other words, the ‘relative’ size is important for DRG neurons.

Finally, even though it can be considered that Cluster 1 DRG neurons, probably corresponding to Aβ-fiber neurons, may release OXT following to mechanical stimulation, such as adhesion of pyramidal thorn patches [21], regardless of nociception in the DRG, direct evidence about the release of OXT and the physiological function of OXT in the DRG has not been demonstrated. Thus, in the next study, we should show that mechanoreception and nociception themselves induce the autocrine/paracrine function of OXT in the DRG, and that OXT interferes the pain signal.

## Data Availability

All of the data generated and analyzed in this study are included in this published article.

## Abbreviations

DRG: Dorsal root ganglion
OXT: Oxytocin
OXTR: OXT receptor
Piezo2: Piezo-type mechanosensitive ion channel component 2
TRPV1: Transient receptor potential cation channel subfamily V member 1
V1aR: Vasopressin V1a receptor

## References

[1] Kandel ER, Schwartz JH, Jessell TM. Principles of Neural Science. In: Chap. 24: The perception of pain. 4th edn, New York: McGraw-Hill; 2000. p. 472–491.

[2] Meltzer S, Santiago C, Sharma N, Ginty DD (2021). The cellular and molecular basis of somatosensory neuron development. Neuron.

[3] Graham RD, Sankarasubramanian V, Lempka SF (2022). Dorsal root ganglion stimulation for chronic pain: Hypothesized mechanisms of action. J Pain.

[4] Wang F, Stefano GB, Kream RM (2014). Epigenetic modification of DRG neuronal gene expression subsequent to nerve injury: etiological contribution to complex regional pain syndromes (Part II). Med Sci Monit.

[5] Saito N, Shima R, Yamada Y, Nagaoka M, Ito E, Yoshioka T (2018). A Proposed molecular mechanism for physical analgesia in chronic pain. Neural Plast.

[6] Ito E, Shima R, Yoshioka T (2019). A novel role of oxytocin: Oxytocin-induced well-being in humans. Biophys Physicobiol.

[7] Hyodo S. 8 C: Oxytocin. In: Ando H, Ukena K, Nagata S. Handbook of hormones. 2nd edn, London: Elsevier (2021).

[8] Qiu F, Qiu CY, Cai H, Liu TT, Qu ZW, Yang Z, Li JD, Zhou QY, Hu WP (2014). Oxytocin inhibits the activity of acid-sensing ion channels through the vasopressin, V1A receptor in primary sensory neurons. Br J Pharmacol.

[9] Gong L, Gao F, Li J, Li J, Yu X, Ma X, Zheng W, Cui S, Liu K, Zhang M, Kunze W, Liu CY (2015). Oxytocin-induced membrane hyperpolarization in pain-sensitive dorsal root ganglia neurons mediated by Ca^2+^/nNOS/NO/KATP pathway. Neuroscience.

[10] Dayanithi G, Forostyak O, Forostyak S, Kayano T, Ueta Y, Verkhratsky A (2018). Vasopressin and oxytocin in sensory neurones: expression, exocytotic release and regulation by lactation. Sci Rep.

[11] Han RT, Kim HB, Kim YB, Choi K, Park GY, Lee PR, Lee J, Kim HY, Park CK, Kang Y, Oh SB, Na HS (2018). Oxytocin produces thermal analgesia via vasopressin-1a receptor by modulating TRPV1 and potassium conductance in the dorsal root ganglion neurons. Korean J Physiol Pharmacol.

[12] Li CL, Li KC, Wu D, Chen Y, Luo H, Zhao JR, Wang SS, Sun MM, Lu YJ, Zhong YQ, Hu XY, Hou R, Zhou BB, Bao L, Xiao HS, Zhang X (2016). Somatosensory neuron types identified by high-coverage single-cell RNA-sequencing and functional heterogeneity. Cell Res.

[13] Yang Q, Wu ZZ, Li X, Li ZW, Wei JB, Hu QS (2002). Modulation by oxytocin of ATP-activated currents in rat dorsal root ganglion neurons. Neuropharmacology.

[14] Moreno-López Y, Martínez-Lorenzana G, Condés-Lara M, Rojas-Piloni G (2013). Identification of oxytocin receptor in the dorsal horn and nociceptive dorsal root ganglion neurons. Neuropeptides.

[15] Ito E, Ikemoto Y, Yoshioka T (2015). Thermodynamic implications of high *Q*_10_ of thermo-TRP channels in living cells. Biophysics.

[16] Nersesyan Y, Demirkhanyan L, Cabezas-Bratesco D, Oakes V, Kusuda R, Dawson T, Sun X, Cao C, Cohen AM, Chelluboina B, Veeravalli KK, Zimmermann K, Domene C, Brauchi S, Zakharian E (2017). Oxytocin modulates nociception as an agonist of pain-sensing TRPV1. Cell Rep.

[17] Michel N, Narayanan P, Shomroni O, Schmidt M (2020). Maturational changes in mouse cutaneous touch and Piezo2-mediated mechanotransduction. Cell Rep.

[18] Horn HS (1966). Measurement of “overlap” in comparative ecological studies. Am Nat.

[19] Rempala GA, Seweryn M (2013). Methods for diversity and overlap analysis in T-cell receptor populations. J Math Biol.

[20] Legendre P, Legendre L. Numerical ecology. In: Chap. 8: Cluster analysis. 3rd edn. Amsterdam: Elsevier; 2012. p. 373–424.

[21] Saito N, Shima R, Yen CT, Yang RC, Ito E, Yoshioka T (2019). Adhesive pyramidal thorn patches provide pain relief to athletes. Kaohsiung J Med Sci.

[22] Owens DM, Lumpkin EA (2014). Diversification and specialization of touch receptors in skin. Cold Spring Harb Perspect Med.

[23] Zimmerman A, Bai L, Ginty DD (2014). The gentle touch receptors of mammalian skin. Science.

[24] Melzack R, Wall PD (1965). Pain mechanisms: a new theory. Science.

[25] Mendell LM (2014). Constructing and deconstructing the gate theory of pain. Pain.

[26] Breton JD, Veinante P, Uhl-Bronner S, Vergnano AM, Freund-Mercier MJ, Schlichter R, Poisbeau P (2008). Oxytocin-induced antinociception in the spinal cord is mediated by a subpopulation of glutamatergic neurons in lamina I–II which amplify GABAergic inhibition. Mol Pain.

[27] Condés-Lara M, Rojas-Piloni G, Martínez-Lorenzana G, López-Hidalgo M, Rodríguez-Jiménez J (2009). Hypothalamospinal oxytocinergic antinociception is mediated by GABAergic and opiate neurons that reduce A-delta and C fiber primary afferent excitation of spinal cord cells. Brain Res.

[28] Mazzuca M, Minlebaev M, Shakirzyanova A, Tyzio R, Taccola G, Janackova S, Gataullina S, Ben-Ari Y, Giniatullin R, Khazipov R (2011). Newborn analgesia mediated by oxytocin during delivery. Front Cell Neurosci.

[29] Schorscher-Petcu A, Sotocinal S, Ciura S, Dupré A, Ritchie J, Sorge RE, Crawley JN, Hu SB, Nishimori K, Young LJ, Tribollet E, Quirion R, Mogil JS (2010). Oxytocin-induced analgesia and scratching are mediated by the vasopressin-1A receptor in the mouse. J Neurosci.

[30] McGlone F, Wessberg J, Olausson H (2014). Discriminative and affective touch: sensing and feeling. Neuron.

[31] Ito E, Oka K, Koshikawa F (2022). Dorsolateral prefrontal cortex sensing analgesia. Biophys Physicobiol..

[32] Le Pichon CE, Chesler AT (2014). The functional and anatomical dissection of somatosensory subpopulations using mouse genetics. Front Neuroanat.

